# A four-long non-coding RNA signature in predicting breast cancer survival

**DOI:** 10.1186/s13046-014-0084-7

**Published:** 2014-10-06

**Authors:** Jin Meng, Ping Li, Qing Zhang, Zhangru Yang, Shen Fu

**Affiliations:** Department of Radiation Oncology, Shanghai Jiao Tong University Affiliated Sixth People’s Hospital, 600 Yi Shan Rd, Shanghai, 200233 China; Radiation Oncology Center, Fudan University Shanghai Cancer Center (FUSCC), 399 LingLing Rd, Xu Hui District Shanghai, 200032 China; Radiation Oncology Department, Shanghai Proton and Heavy Ion Center (SPHIC), 4365 Kang Xin Rd, Pudong New District Shanghai, 201321 China

**Keywords:** Long non coding RNA, lncRNA, Breast cancer, Prognostic, Biomarker, Expression, Gene signature, Survival

## Abstract

**Background:**

Many long non-coding RNAs(lncRNAs) have been found to be a good marker for several tumors. Using lncRNA-mining approach, we aimed to identify lncRNA expression signature that can predict breast cancer patient survival.

**Methods:**

We performed LncRNA expression profiling in 887 breast cancer patients from Gene Expression Omnibus (GEO) datasets. The association between lncRNA signature and clinical survival was analyzed using the training set(n = 327, from GSE 20685). The validation for the association was performed in another three independent testing sets(252 from GSE21653, 204 from GSE12276, and 104 from GSE42568).

**Results:**

A set of four lncRNA genes (U79277, AK024118, BC040204, AK000974) have been identified by the random survival forest algorithm. Using a risk score based on the expression signature of these lncRNAs, we separated the patients into low-risk and high-risk groups with significantly different survival times in the training set. This signature was validated in the other three cohorts. Further study revealed that the four-lncRNA expression signature was independent of age and subtype. Gene Set Enrichment Analysis (GSEA) suggested that gene sets were involved in several cancer metastasis related pathways.

**Conclusions:**

These findings indicate that lncRNAs may be implicated in breast cancer pathogenesis. The four-lncRNA signature may have clinical implications in the selection of high-risk patients for adjuvant therapy.

**Electronic supplementary material:**

The online version of this article (doi:10.1186/s13046-014-0084-7) contains supplementary material, which is available to authorized users.

## Background

More than 50% of transcripts have no protein coding potential through the analysis of mammalian transcriptomes, a subset of these noncoding transcripts are termed long non-coding RNAs (lncRNAs) that range from 200 nucleotides to multiple kilobases in length [1]. These long, polyadenylated RNAs do not code for proteins, but function directly as RNAs. Many lncRNAs have already been associated with various disease processes, and cancer features prominently among these. In addition to the classic protein coding mRNAs, recent studies have revealed the contribution of lncRNAs as protooncogenes, tumor suppressor genes, and drivers of metastatic transformation at the transcriptional, post-transcriptional, and epigenetic levels [2-5]. Accumulating evidence indicates that lncRNAs are linked to a diverse range of functions in cellular development and their misregulation has also been implicated in various types of cancers [6-8]. In most cases, these transcripts are aberrantly expressed in cancers, which may indicate their potential as possible biomarkers and can be predictive of clinical outcome.

Breast cancer is a heterogeneous disease composed of multiple molecular alterations. Molecular differences between histologically similar tumors make clinical outcomes difficult to predict and treatment imperfectly adapted [9]. Breast cancers of varying histological subtypes and risk stratification are traditionally diagnosed based on their histopathological features, including tumor size, grade and lymph node status. Over the past decade, the “intrinsic” molecular subtypes of breast cancer: luminal A and B, basal, ERBB2 and normal-like, exhibit different histo-clinical features and treatment sensitivity [10,11]. Given the heterogeneity of breast cancer and the multitude of variables influencing clinical evolution, the multi-gene signatures provide further prognostic and predictive information. One of the examples is a 21-gene classifer (Oncotype DX) ,which classifies breast tumors into low-,intermediate- and high-risk groups as to the advisability of adjuvant chemotherapy for patients in high-risk group [12,13]. The utility of such gene signature might have clinical potential to predict patient outcome and aid in treatment choice [14].

In breast cancer, several lncRNA transcripts were involved in the biology of tumorigenesis. Furthermore, certain lncRNAs exhibit distinct expression patterns between primary tumors and metastases. A 2.2 kb lncRNA, HOTAIR has been shown to be an independent predictor of breast cancer survival. Elevated HOTAIR expression levels correlate with breast cancer, and are linked to poor prognosis and metastasis [3]. This lncRNA may induce metastases by remodeling the epigenetic machinery to repress metastasis suppressor genes (e.g., HOXD10 ). Another lncRNA, MALAT-1 (metastasis associated lung adenocarcinoma transcript 1) is overexpressed in many different cancer types, including lung, breast, colon, prostate, pancreatic, and hepatocellular carcinomas [15-17]. This highly conserved 8kb lncRNA is upregulated in invasive breast carcinomas and correlates with tumor grade [18]. GAS5 (growth arrest-specific 5) was found to be downregulated in breast cancer tissues, and overexpression of this lncRNA in the MCF-7 breast cancer cell line furthered growth arrest and apoptosis [19]. LSINCT5, the stress-regulated lncRNA, is overexpressed in breast and ovarian cancer cell lines and tumor tissues. In addition, LSINCT5 has been proved to play a role in cellular proliferation and also in the development of breast and ovarian cancers [20]. Transcriptional profiling has revealed highly aberrant lncRNA expression in human cancers [21]. However, the prognostic significance of lncRNAs in breast cancer has not been investigated.

Recently, the methodology of repurposing microarray data for probing lncRNA expression is well-established [22-24]. For instance, Du et al. used a large dataset of microarrays to build a resource of clinically relevant lncRNAs for the development of lncRNA biomarkers and the identification of lncRNA therapeutic targets [25]. Zhang et al. correlates lncRNA expression profiles with malignancy grade and histological differentiation in human gliomas by re-annotating Affymetrix HG-U133 Plus 2.0 array [26,27]. Furthermore, several studies do have discovered new biomarkers to predict survival by re-annotation of previous microarray data. A six-lncRNA signature has been identified to predict survival of patients with glioblastoma multiforme [27], while a three-lncRNA signature has been shown to be associated with the prognosis of patients with oesophageal squamous cell carcinoma [28].

In this study, we aimed at profiling the lncRNA expression signatures by analyzing a cohort of previously published breast cancer gene expression profiles from the Gene Expression Omnibus (GEO), as well as another three independent data sets as testing sets. We identified a four-lncRNA signature associated with survival, and then established a risk score formula using the expressions of these four lncRNAs. The prognostic value of the signature was further confirmed in the testing cohorts. Our findings suggest that lncRNA signatures can be predictive of clinical outcome and they may be useful as biomarkers.

## Materials and methods

### GEO breast cancer gene expression data

**Figure 1:**
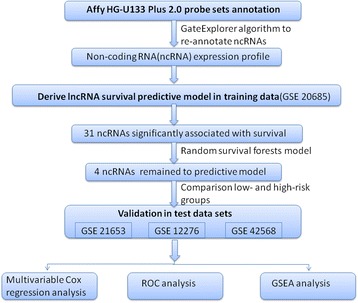
**Diagram of the study.** The order of analyses to develop the risk score model and validate the efficiency of the gene signature to predict prognostic outcomes.

Breast cancer gene expression data and corresponding clinical data used in this study were obtained from the publicly available GEO databases. To analyze the correlation of lncRNA expression signatures with survival endpoints for breast cancer as a whole (disease-free survival, metastasis-free survival and overall survival), we selected those data sets that included more than 100 patients with their survival status information. We followed the strategy of using the largest data set (GSE20685) as training set. This training set from GSE20685 [29] was first used to identify the gene expression signature. Another three independent data sets from GSE12276 [30], GSE21653 [31,32], GSE42568 [33] were included in this study as testing sets. After filtering out samples without clinical survival information, there were a total of 887 samples, including 327 from GSE20685, 252 from GSE21653, 204 from GSE12276, and 104 from GSE42568, respectively. Figure 1 depicts the diagram of the study.

### Microarray data processing and LncRNA profile mining

The raw CEL files were downloaded from GEO database and background adjusted using Robust Multichip Average. GATExplorer [22] was used to process microarrays on a local computer for gene expressions of lncRNAs. This GATExplorer provides a series of R packages, designed to be used with BioConductor tools, that allow to apply in a simple way the probe mapping data included in GATExplorer. A type of files called ncRNA Mapper were also obtained from GATExplorer, which include the probes that do not map to any coding region but that were mapped to a database for non-coding RNA of human and mouse (derived from RNAdb [34]). A customized R scripts was used to perform a microarray expression calculation according to the re-mapping data(file ncrnamapperhgu133plus2cdf_3.0). Each LncRNA should include at least a minimum of 3 probes mapping in the corresponding ncRNAs entity. All of the four lncRNAs were verified online in the ncRNA Expression Database (nred.matticklab.com) [35], which provides gene expression information for thousands of long ncRNAs in human and mouse (Additional file 1: Table S1). We created a risk-score formula according to the expressions of these four lncRNAs for survival prediction. Patients having higher risk scores are expected to have poorer survival outcomes. The risk scores are calculated as follows: Risk score = (−0.35717× expression value of AK024118) + (0.518242 × expression value of U79277) + (−0.48664 × expression value of BC040204) + (−0.48122 × expression value of AK000974). In addition, the coding potential analysis of the lncRNAs was carried out by CNCI to classify protein-coding or noncoding transcripts [36].

### Gene set enrichment analysis (GSEA)

GSEA was performed by the JAVA program (http://www.broadinstitute.org/gsea) using MSigDB C2 CP: Canonical pathways gene set collection(1320 gene sets available). Gene sets with a false discovery rate(FDR) value <0.05 after performing 1,000 permutations were considered to be significantly enriched [37]. Cytoscape and Enrichment Map were used for visualization of the GSEA results.

### Statistical analysis

**Figure 2:**
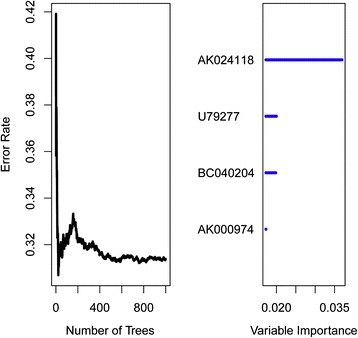
**Error rate for the data as a function of trees (left) and out-of-bag importance values for predictors (right).**

The association between the lncRNA gene expression and patient’s survival was assessed by univariable Cox regression analysis along with a permutation test using Biometric Research Branch-Array tools package [38] in the training set. With a parametric test(p ≤ 0.001), we identified a set of 30 lncRNA expressions strongly correlated with survival. Considering that a smaller number of genes in the model would make the model more practical, we then performed the random survival forests-variable hunting (RSFVH) algorithm [39]. We followed Kawaguchi [39,40] for the parameters in the algorithm. In brief, the number of Monte Carlo iterations (nrep) was set as n_rep_ = 100 and value controlling the step size used in the forward process (nstep) was set as n_step_ = 5. A set of 4 lncRNAs genes have been identified in which expressions were strongly and consistently related to patient survival (Figure 2).

Using these four genes selected fitted in a multivariable Cox regression model, we constructed a formula that would predict survival in the training set. Each patient was then assigned a risk score that is a linear combination of the expression levels of the significant lncRNAs weighted by their respective Cox regression coefficients [41,42]. According to this risk score, patients in the training set were divided into low-risk and high-risk groups using the median risk score as the cut-off. The Kaplan-Meier method was used to estimate survival time for the other three testing groups. Differences in survival times between the low-risk and high-risk groups in each set were then compared using the two-sided log rank test. Furthermore, we also used Cox multivariate analysis to test whether the risk score was independent of patient age and subtype with the available data. Oncotype DX score was implemented in an R package called genefu, available from the Comprehensive R Archive Network. We used receiver operating characteristic (ROC) curves to compare the sensitivity and specificity of the survival prediction of the lncRNA risk score. Area under the curve (AUC) values were calculated from the ROC curves [43]. All the data were analyzed by R program (www.r-project.org). The significance was defined as p values being less than 0.05.

## Results

### Identification of lncRNA genes from the training set

As summarized in the workflow (Figure 1), all analyses were performed in the training data set (GSE20685) first and then validated in the test data set (GSE21653, GSE12276, GSE42568). The training set(n = 327) was analyzed for the detection of prognostic lncRNA genes. By subjecting the lncRNA expression data derived from the training set to univariable Cox proportional hazards regression analysis using the BRB-Array Tools, we identified a set of 30 lncRNAs that were strongly correlated with patients’ overall survival (P ≤ 0.001). On the basis of the random survival forests model (see Materials and methods), four genes were selected as the predictors. Table 1 shows a list of these four genes with their obtained variable importance values. As depicted in Figure 2, from the plot we can see that AK024118 has dramatically larger importance value than other predictors. Of these, a positive coefficient of U79277 indicated that its higher level of expression was associated with shorter survival. The negative coefficients of the other genes (AK024118, BC040204, AK000974) indicated that their higher levels of expression were associated with longer survival. All of the four lncRNAs have been verified in the ncRNA Expression Database (nred.matticklab.com) and these four transcripts were classified as ncRNAs in this website [35]. As coding potential analysis is commonly used to classify whether a transcript is of coding potential or not [25], we also used another tool, CNCI, developed by Sun et.al to test those four transcripts [36]. This tool also suggests that all the four transcripts are non-coding transcripts with no coding potential.

**Table 1:** **Four LncRNAs significantly associated with the overall survival in the training-set patients (n = 327)**

**Gene symbol**	**Chromosomal position**	**Parametric P value**	**Hazard ratio**	**Coefficient**	**Variable importance**	**Relative importance**
AK024118	chr18:59125236-59125296	7.00E-07	0.579	−0.35717	0.0369	1.0000
BC040204	chr6:72153349-72153409	1.93E-05	0.428	−0.48664	0.0198	0.5368
AK000974	chr10:97810995-97811055	2.85E-05	0.416	−0.48122	0.0173	0.4683
U79277	chr8:101998264-101998324	4.30E-06	2.17	0.518242	0.0200	0.5411

### The association of four-lncRNA signature and patient’s survival in the training set

**Figure 3:**
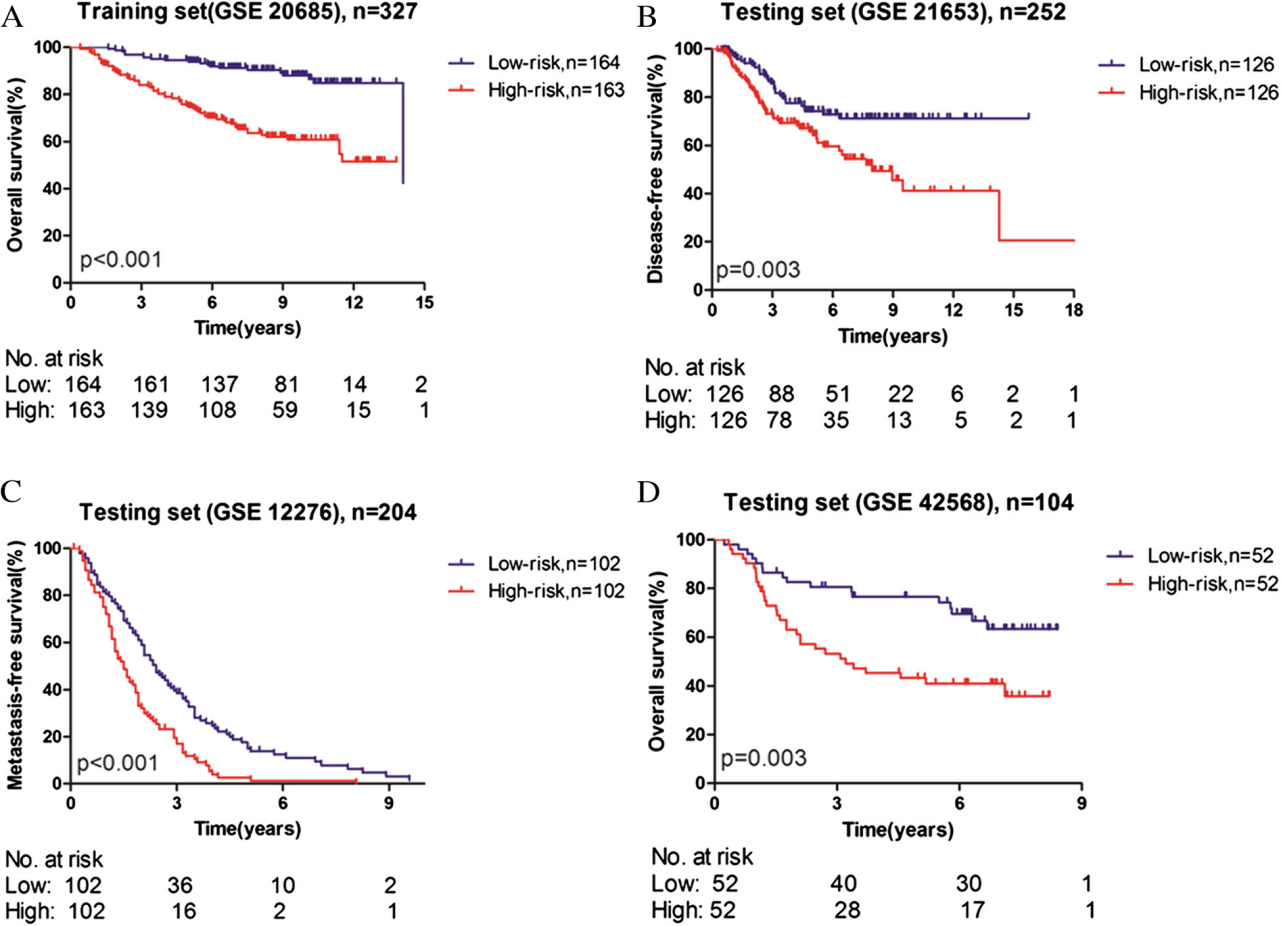
**Kaplan–Meier estimates of the survival of Gene Expression Omnibus (GEO) patients using the four-lncRNA signature.** The Kaplan–Meier plots were used to visualize the survival probabilities for the low-risk versus high-risk group of GEO patients determined on the basis of the median risk score from the training set patients. **(A)** Kaplan–Meier curves for GSE20685 training-set patients (n = 327); **(B)** Kaplan–Meier curves for GSE21653 testing-set patients (n = 252); **(C)** Kaplan–Meier curves for GSE12276 testing-set patients (n = 204); **(D)** Kaplan–Meier curves for GSE42568 testing-set patients (n = 104). The tick marks on the Kaplan–Meier curves represent the censored subjects. The differences between the two curves were determined by the two-sided log-rank test. The number of patients at risk was listed below the survival curves.

With the risk score formula (see Materials and methods), we calculated the four-lncRNA expression signature risk score for each patient in the training set. The patients were then ranked according to their risk scores. Using the median risk score as cut-off in the training set, the patients were divided into low-risk(n = 164) and high-risk (n = 163) groups. Patients in the high-risk group had significantly shorter overall survival than those in the low-risk group (log-rank test P < 0.0001) (Figure 3A). Overall survival in the training set was 96.95% at 3 years, 91.89% at 6 years, 88.08% at 9 years and 84.86% at 12 years in the low risk group, versus 83.99%, 70.27%, 62.02% and 51.56% in the high risk group respectively. The correlation of the four-lncRNA risk score with overall survival was significant when it was analyzed as a continuous variable in the univariable Cox regression model.

### Validation of the four-lncRNA signature for survival prediction in the testing sets

**Figure 4:**
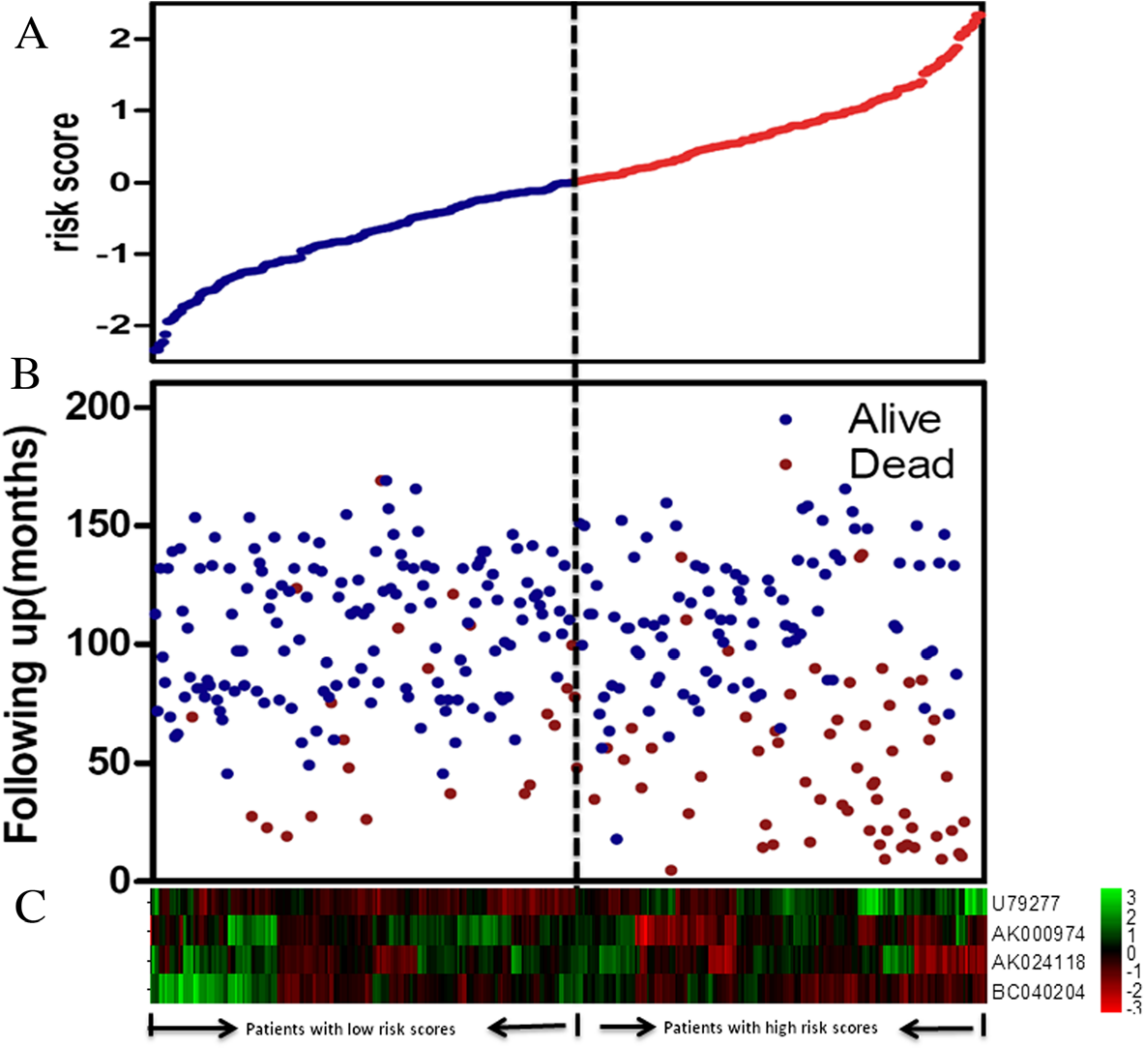
**LncRNA risk score analysis of GEO patients.** The distribution of four-lncRNA risk score, patients’ survival status and lncRNA expression signature were analyzed in the training set patients (n = 327). **(A)** LncRNA risk score distribution; **(B)** patients’ overall survival status and time; **(C)** heatmap of the lncRNA expression profiles. Rows represent lncRNAs, and columns represent patients. The black dotted line represents the median lncRNA risk score cutoff dividing patients into low-risk and high-risk groups.

In order to confirm our findings, we calculated the risk score for the testing sets including GSE21653(n = 252), GSE12276 (n = 204) and GSE42568(n = 104). By using the same cut-off value as the training set, the patients from each testing set were separately classified into low-risk and high-risk groups and subjected to survival comparison. As overall survival information was unavailable in GSE21653 and GSE12276, disease-free survival(DFS) and metastasis-free survival(MFS) was evaluated, respectively. Similar to the findings obtained from the training set, patients in the high-risk group had shorter survival time than patients in the low-risk group (Figures 3B, 3C, 3D). In consistence with the results described above, patient survival in the low-risk group was better than that in the high-risk group throughout the follow-up. In the univariable Cox regression model, the similar correlation of the risk score with overall survival was noted with the high-risk group having a shorter overall survival than the low-risk group. The distribution of patient risk scores (Z-score transformed), survival status and lncRNA values were analyzed independently for the training set (Figure 4). We found that patients with high-risk scores tended to have higher expression of U79277 and lower expression of the remaining genes(AK024118, BC040204, AK000974). Detail survival information of individual lncRNA in each data set and the gene signature in the context of different tumor subtypes was shown in Additional file 2: Figure S1 and Additional file 3: Figure S2, respectively.

### Multivariate regression analysis shows that the four-lncRNA expression signature is independent of age and subtype

We carried out Cox multivariate analysis to ascertain whether the four-lncRNA expression signature was an independent predictor of breast cancer patient’s survival. Four-lncRNA risk score, age (available in GSE20685, GSE21653 and GSE42568) and subtype(only available in GSE21653) were defined as covariates. The effect of risk score, age and subtype on breast cancer patient survival time was further evaluated by multivariate Cox proportional hazard model. The results showed that risk score is an independent predictor of patient survival when adjusted by age or subtype in every cohort (Table 2).

**Table 2:** **Univariable and multivariable Cox regression analyses in each data set**

**Variables**	**Univariable model**			**Multivariable model**		
	**HR**	**95% CI of HR**	**p value**	**HR**	**95% CI of HR**	**p value**
Training set (GSE 20685) (N = 327)						
Four-lncRNA risk score	3.89	2.33-6.51	2.10E-8	3.88	2.32-6.48	2.30E-7
Age	0.99	0.97-1.01	0.49	0.99	0.97-1.02	0.62
Testing set (GSE 21653) (N = 252)						
Four-lncRNA risk score	2.12	1.35-3.34	0.001	2.28	1.44-3.63	4.84E-4
Age	1.00	0.98-1.02	0.95	1.00	0.98-1.02	0.88
Subtype	1.04	0.88-1.23	0.64	1.12	0.95-1.33	0.19
Testing set (GSE 12276) (N = 204)^a^						
Four-lncRNA risk score	1.893	1.40-2.56	2.33E-5			
Testing set (GSE 42568) (N = 104)						
Four-lncRNA risk score	2.36	1.30-4.27	0.005	2.34	1.29-4.24	0.005
Age	1.00	0.97-1.02	0.59	1.00	0.97-1.02	0.78

^a^In GSE12276 set, there was no available age or subtype information.

### Evaluation of the risk score performance by receiver operating characteristic (ROC) curve analysis

**Figure 5:**
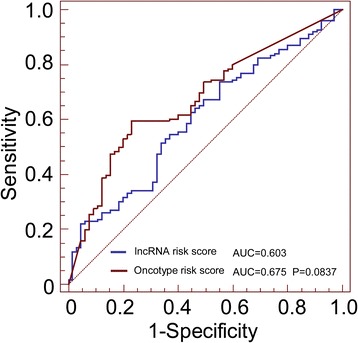
**Receiver operating characteristic (ROC) analysis of sensitivity and specificity by four-lncRNA risk score, oncotype in predicting disease-free survival (DFS).** In GSE21653, the score performance was assessed by calculating the area under the ROC (AUROC) of four-lncRNA risk score versus Oncotype, which was 0.603 and 0.675, respectively. (p = 0.0837).

As GSE21653 was the only data set with disease-free survival information(DFS), we performed receiver operating characteristic (ROC) analysis to compare the sensitivity and specificity of survival prediction between our model and Oncotype DX [13]. The area under receiver operating characteristic (AUROC) was determined and compared between these two gene signatures. As seen in Figure 5, ROC curves indicated that AUROC of four-lncRNA gene signature and Oncotype DX was 0.603 and 0.675, respectively. No significant difference(p=0.0837) was observed between the Oncotype group and the four-lncRNA gene signature group in terms of disease-free survival (DFS) (Figure 3).

### Identification of four-lncRNA signature associated biological pathways and processes

**Figure 6:**
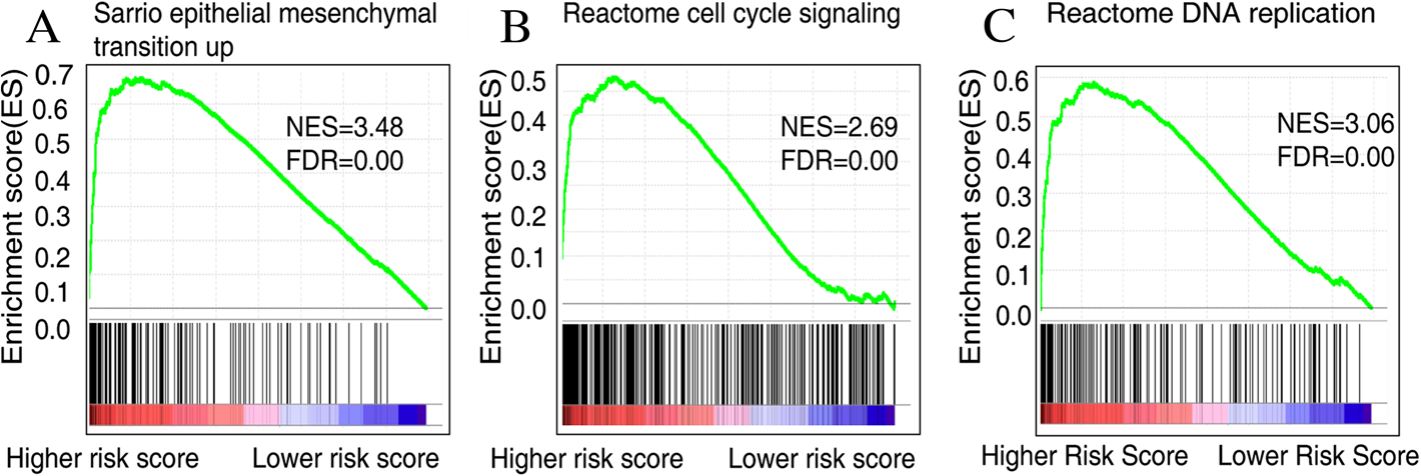
**Gene set enrichment analysis delineates biological pathways and processes associated with risk score in the training set(GSE 20685).** GSEA validated enhanced activity of **(A)** epithelial mesenchymal transition pathway **(B)** cell cycle signaling pathway **(C)** DNA replication pathway in high risk score group.

Gene Set Enrichment Analysis(GSEA) was carried out to identify associated biological processes and signaling pathway [37]. We compared the gene expression profile of breast cancer patients with high-risk and low-risk group classified by four-lncRNA gene signature in the training set(GSE 20685). The gene sets with significantly different expression (FDR < 0.01, p <0.005) were picked up for Gene set enrichment analysis (GSEA). Several cancer related pathways such as epithelial mesenchymal transition(EMT) [44], cell cycle signaling and DNA replication [45] were enriched in the high risk group, which implies that the signature might be involved in the metastasis related pathways (Figure 6). The associated biological pathway with each lncRNA was shown in Additional file 4: Figure S3.

## Discussion

The discovery of multiple functional regulatory lncRNAs has lead to genome-wide searches in multiple species as well as for transcripts that are aberrantly expressed in various types of cancers. Similar to protein-coding genes and miRNAs, lncRNAs play key roles in tumorigenesis. They are involved in a number of fundamental processes associated with cancer including cell cycle regulation, apoptosis, the DNA damage response, and metastasis [3,46]. The expression of highly conserved lncRNAs is also altered in breast cancers [17]. Our recent study achieved the lncRNA profiling by mining the existing microarray gene expression data as reported [47,48]. Except for several recent researches on the roles of lncRNAs in breast cancer, the prognostic value of lncRNA signatures have not been investigated. To our knowledge, this is the first report of a lncRNA expression signature predicting breast cancer patient survival.

In this study, we have identified a four-lncRNA expression signature that is associated with survival of breast cancer patients. We further revealed that the four-lncRNA signature is an independent predictor of breast cancer patient survival.

As for the characteristics of the four genes, the overexpression of U79277 was found to be correlated with shorter survival while three of these four lncRNAs identified (AK024118, BC040204, AK000974) were downregulated in the high-risk group compared to low-risk group. The functional study in cancer of these genes has not been reported so far. Nevertheless, our present study demonstrated the associations between the expressions of these genes and survival time. Interestingly, the locations of those putative lncRNAs overlap with many transcripts including some well-known oncogene and tumor suppress genes. AK024118 is located within the intron of BCL2 which is a known driver of lymphoma. U79277 is transcribed from the minus strand on human chromosome 8 and overlaps with YWHAZ 3’UTR. AK000974 which overlaps with many transcripts including CCNJ mRNA. We found that it is very common for ncRNAs. The lncRNAs were categorized as intergenic or genic. The genic lncRNAs were further classified as being exonic, intronic or overlapping and sense or antisense according to their relation with neighboring protein coding genes [49,50]. Although some of lncRNAs may overlap with neighboring protein coding genes, most of them have their own function. Some lncRNAs regulate the transcription of nearby genes in cis, while others act in trans [28]. A concrete example is HOTAIR, a well-studied lncRNA, within the HOXC cluster was shown to help silence HOXD cluster genes in trans [51]. It may be worthwhile to further investigate these lncRNAs for the purpose of better understanding of their roles in determining breast cancer prognosis.

The median risk score was used as a cutoff point for two reasons. First, a previous lncRNA risk score formula used the median as a cutoff point for classifying patients into two groups [27]. Second, the most common approach for dichotomizing continuous variables was to take the sample median due to the absence of a prior cutpoint [52].

By performing multivariable Cox regression analysis that included age and subtype(when available) as covariables, we analyzed whether the prognostic value of the four-lncRNA signature was independent of age and subtype. The age at diagnosis exercises a complex influence on breast cancer prognosis. Young age at diagnosis influences negatively the prognosis [53-55], whereas breast cancer in elderly women is associated with an inferior prognosis when compared to that of middle-aged women [54]. Observational data in breast cancer patients is suggestive of an increased risk of disease specific mortality with increasing age [56,57]. Several observations suggest that the percentage of deaths attributed to breast cancer decreased with age [58,59]. These inconsistency in findings could explain the results that age was not significant prognostic factor when assessed in the univariable Cox regression analysis in our study. Nonetheless, we could conclude that the risk score obtained by the four-lncRNA signature was independent of age in the present study.

Breast cancer is clinically heteregeneous due to molecular differences between histologically similar tumors. Luminal, Her2 enriched, basal-like (Triple-negative) subgroups were identified and were shown to have different long-term survivals [10,11,60,61]. There were few reports about the correlation between lncRNAs and molecular subtype of breast cancer. A newly identified lncRNA, LOC554202, has been found to express abundantly in the non-invasive breast cancer cell lines like luminal subtype, but the expression is lost in more aggressive triple-negative breast cancer cell lines of basal subtype [62]. It was therefore of interest to determine if our four-lncRNA signature was associated with this strong prognostic factor. As the data on molecular subtype was only available in GSE21653, we performed the analysis of multivariable Cox regression including risk score and subtype in this testing group. Because of the small sample size in some subgroups, we did not observe significant difference in either univariable or multivariable Cox regression analysis.

Further ROC analysis demonstrated that four-lncRNA gene signature was comparable with Oncotype DX (p = 0.0837). Although Oncotype DX is the most accepted in clinical practice for decision making as to the advisability of adjuvant chemotherapy for breast cancer patients [12], the test is not financially feasible for every patient in developing countries. As shown in this study, a small number of genes (4 genes) could be sufficient to predict the prognostic, using simply reverse transcription polymerase chain reaction (RT-PCR). Clinically, risk score may provide clues on biological behaviors as well as prognostic characteristics of tumors. Patients belonging to high-risk group may need more effective adjuvant therapy in addition to the standard treatment protocol. In addition to the current prognostic model, the four-lncRNA signature may develop easy-to-use prognostic model in order to facilitate further stratification of patients.

Moreover, Gene set enrichment analysis (GSEA) was performed aiming at analyzing coordinate expression changes at a pathway level. The associated molecular pathways, namely, epithelial mesenchymal transition(EMT) [44], cell cycle signaling and DNA replication revealed the four-lncRNA signature might be involved with cancer metastasis. Hence, these findings are likely to be implicated in the development of new targeted anti-cancer therapies. In breast cancer, it has been shown that knock-down of lncRNA HOTAIR with specific siRNAs may limit the metastatic potential of breast cancer cells [3]. The therapeutic potential of targeting regulatory lncRNAs in order to increase the expression of specific genes has also recently emerged [1,63]. The four prognostic lncRNAs may have therapeutic potential as novel molecular targets.

Several limitations to this study need to be acknowledged. First, in our study, only a fraction of human lncRNA (5635 out of 15000+) were included in the analysis. So, the prognostic lncRNA genes identified here may not represent all the lncRNA candidates that are potentially correlated with breast cancer overall survival. Secondly, we lack information on the mechanisms behind the prognostic values of these four lncRNAs in breast cancer, and experimental studies on these lncRNAs might provide important information to further our understanding of their functional roles. Finally, although we recapitulated our findings in three published datasets to the extent possible based on data availability, the signature has not yet been tested prospectively in a clinical trial. Despite these drawbacks, however, the significant and consistent correlation of our four-lncRNA signature with overall survival in several independent data sets indicates that it is a potentially powerful prognostic marker for breast cancer.

## Conclusions

In summary, we have identified a set of four-lncRNA signature, which predicts the overall survival in three independent cohorts. Further analysis revealed that the prognostic value was independent of age and subtype. Clinically, the identification of poor or good prognosis cases may help select the appropriate treatment. The identification of the prognostic lncRNAs indicates the potential roles of lncRNAs in breast cancer pathogenesis. The four-lncRNA signature may have clinical implications as molecular diagnosis markers and therapeutic targets.

## Additional files

Additional file 1: Table S1. The LncRNA list re-annotated by GATExplorer HG_U133_Plus_2 ncRNA Mapper. Additional file 2: Figure S1. Survival information of individual lncRNA in each data set. Additional file 3: Figure S2. The gene signature predicts survival in different tumor subtypes. Additional file 4: Figure S3. The associated biological pathway with each lncRNA.

## Abbreviations

lncRNAs: Long non-coding RNAs
GEO: Gene expression omnibus
GSEA: Gene set enrichment analysis
FDR: False discovery rate
RSFVH: Random survival forests-variable hunting
ROC: Receiver operating characteristic
AUC: Area under the curve
DFS: Disease-free survival
GSEA: Gene set enrichment analysis
EMT: Epithelial mesenchymal transition
RT-PCR: Reverse transcription polymerase chain reaction
