# Supplementary figures and images for: A four-long non-coding RNA signature in predicting breast cancer survival
Source: J Exp Clin Cancer Res. 2014 Oct 6;33(1):84. doi: 10.1186/s13046-014-0084-7 (PMC4198622; doi:10.1186/s13046-014-0084-7)

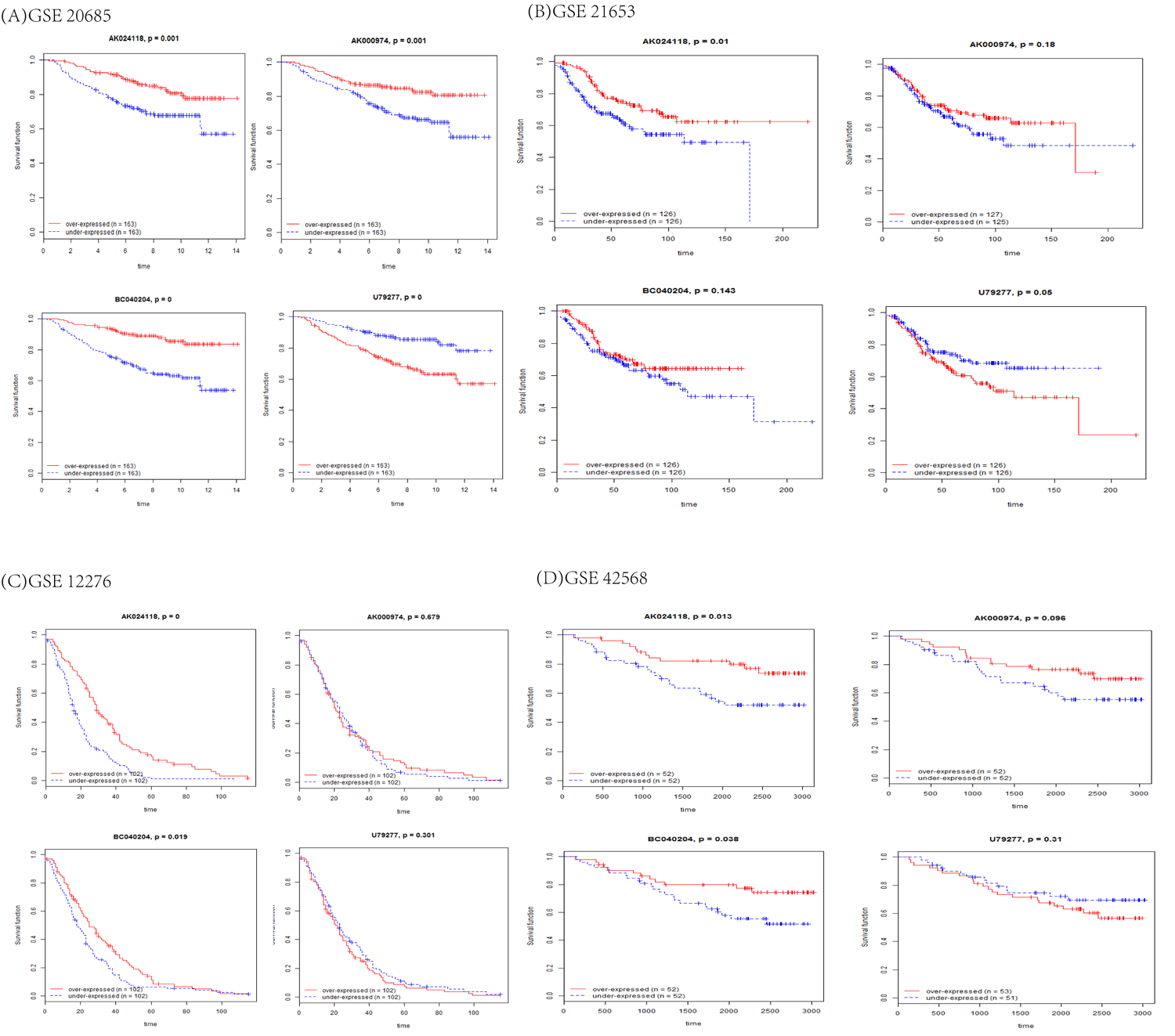

Supplement: Additional file 2: Figure S1. — Survival information of individual lncRNA in each data set. [file 13046_2014_84_MOESM2_ESM.tiff]

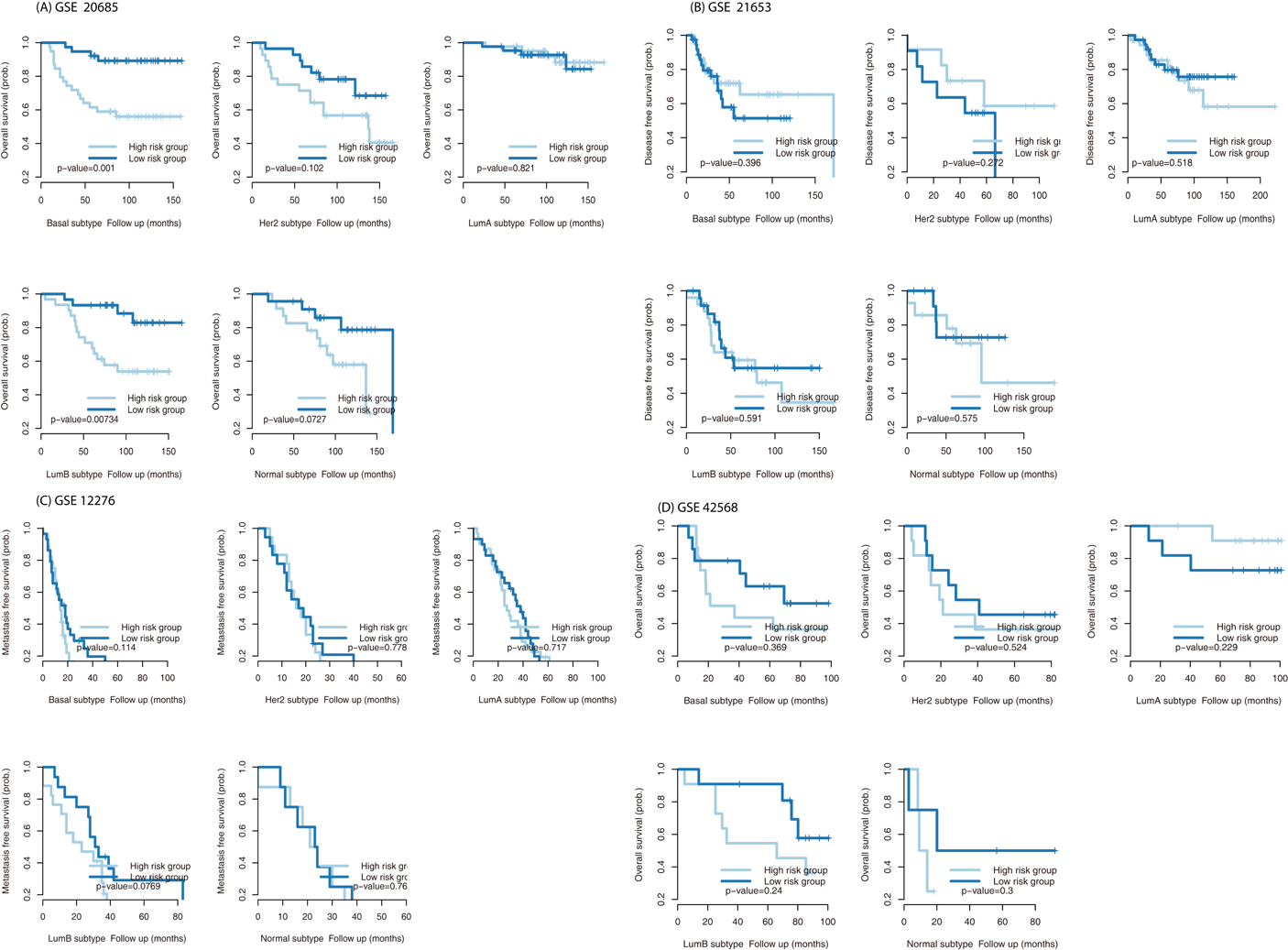

Supplement: Additional file 3: Figure S2. — The gene signature predicts survival in different tumor subtypes. [file 13046_2014_84_MOESM3_ESM.tiff]

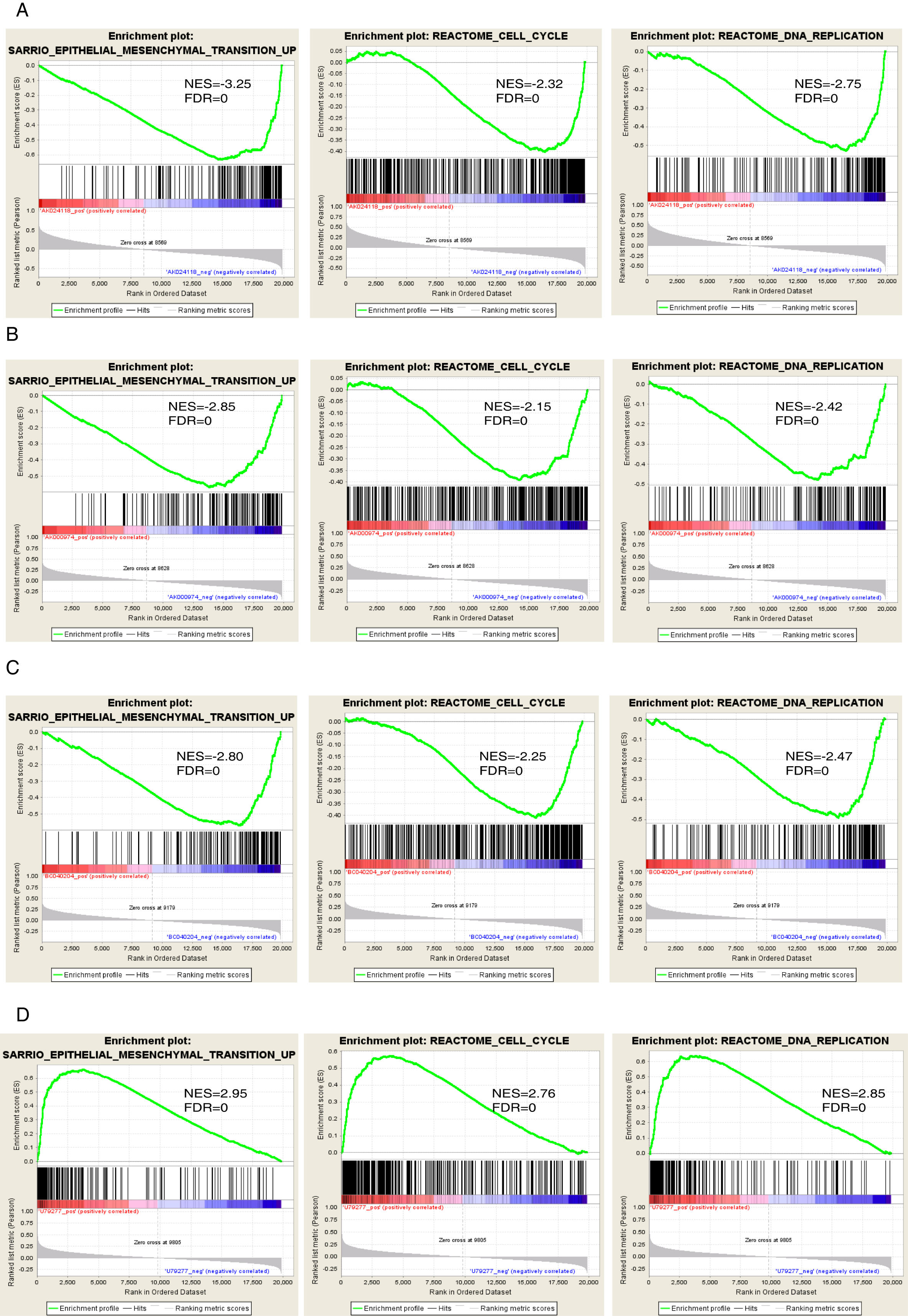

Supplement: Additional file 4: Figure S3. — The associated biological pathway with each lncRNA. [file 13046_2014_84_MOESM4_ESM.tiff]
